# Determining Moisture Condition of External Thermal Insulation Composite System (ETICS) of an Existing Building

**DOI:** 10.3390/ma18030614

**Published:** 2025-01-29

**Authors:** Paweł Krause, Iwona Pokorska-Silva, Łukasz Kosobucki

**Affiliations:** 1 Faculty of Civil Engineering, Silesian University of Technology, 44-100 Gliwice, Poland; pawel.krause@polsl.pl; 2 STEKRA Sp. z o. o., 43-190 Mikołów, Poland; l.kosobucki@stekra.pl

**Keywords:** ETICS, water content, thermal insulation

## Abstract

ETICS is a popular external wall insulation system, which is not without possible defects and damages. A frequent cause, direct or indirect, of damage to buildings is the impact of water (moisture). This article presents, among others, the results of tests of the moisture content of ETICS layers, the water absorption and capillary absorption of the render by means of the Karsten tube method, numerical thermo-moisture simulations, and tests of interlayer adhesion, in sample residential buildings. Mass moisture content testing of the wall substrate showed acceptable moisture levels (1–4%m) within masonry walls made of silicate blocks, as well as locally elevated moisture levels (4–8%m) in the case of reinforced concrete walls. Moisture testing of the insulation samples showed a predominantly dry condition, and testing of the reinforcement layer showed an acceptable level of moisture. Severe moisture was found in the sample taken in the ground-floor zone at the interface between mineral wool and EPS-P insulation underneath the reinforced layer. Capillary water absorption tests helped classify silicone render as an impermeable and surface hydrophobic coating. Tests of the water absorption of the facade plaster showed that the value declared by the manufacturer (<0.5 kg/m^2^) was mostly met (not in the ground-floor zone). The simulation calculations gave information that there was no continuous increase in condensation during the assumed analysis time (the influence of interstitial condensation on the observed anomalies was excluded). The tests carried out indicated the occurrence of numerous errors in the implementation of insulation works affecting the moisture content and durability of external partitions.

## 1. Introduction

The External Thermal Insulation Composite System (ETICS) is one of the most popular methods of insulating the external walls of both newly constructed buildings and existing buildings.

ETICS has been used in European countries since the second part of the last century. Despite the passing years, its use has recently increased significantly due to its functional properties, relatively low execution costs, and successively improving thermal performance [1]. The basic idea of the method involves placing an insulation layer and a facade layer on a suitably prepared substrate. The layout of individual ETICS layers is precisely defined within the national guidelines related to the harmonised technical specification [2]. Additionally, all elements of the system should retain their properties throughout the intended lifespan of the ETICS.

ETICS contributes not only to improving the visual appearance of the building facade but also to improving its energy efficiency [3,4,5] and even, under certain conditions, to improving its acoustic performance [6]. The application of ETICS is also associated with certain faults and disadvantages [7,8,9,10], including staining and the occurrence of microbiological contamination of the building facade [11,12,13,14,15], loss of adhesion between system components [16], or poor impact resistance [17]. ETICS is constantly exposed to a variety of destructive influences that negatively affect its durability [18,19,20]. The most damaging factor could be exposure to water. The dampness of the building envelope is considered to be one of the most common causes of defects in buildings, both in the finishing layer and in structural elements [21,22].

The authors of [20] analysed the effects of moisture and temperature on the ETICS, resulting in internal stresses and frost damage. A study of the hygrothermal behaviour of ETICS is presented in [23], while the moisture transport properties (capillary water absorption, water vapour permeability, low-pressure water absorption, and drying kinetics), thermal conductivity, mould susceptibility, and surface properties for twelve ETICSs were assessed and discussed in [24]. Pereira et al. [21] analysed the influence of moisture on facade cladding degradation in existing buildings, with the conclusion that the ETICS had the highest incidence of moisture-related defects.

Damage (defects) manifests in many cases of facade insulation with an ETICS, potentially compromising the integrity of the system and, in selected cases, resulting in the need to dismantle the system [25].

In order to assess the technical condition of the partition layers, it is necessary to conduct detailed analyses. There are many tools available for assessing the moisture content of building partitions, including computer tools [26,27,28].

This article focuses on a number of anomalies occurring on the facades of exemplary buildings. It raises a question of the source of their occurrence. The causes of local plaster defects and separations could be related to moisture occurring within the wall insulation system, due to design, execution, or material defects. The aim of this article is to answer the question about the sources of irregularities, and to analyse their effects, e.g., the impact of water at the junction of system layers.

## 2. Methodology

**Figure 1:**
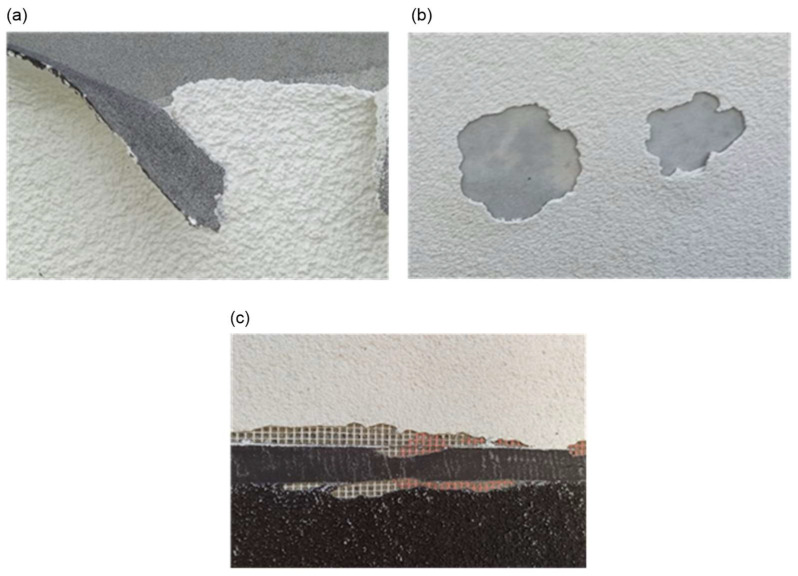
(**a**) Extensive detachment of thin-coat plaster from the reinforced layer along the unplastered strip separating plasters of different colour. (**b**) Fragment of the north elevation of the building in the ground-floor area—detachment and loss of thin-coat plaster. (**c**) Fragment of the north elevation of the building—spalling and loss of thin-coat plaster with exposed reinforcing mesh.

For the analysis, buildings were selected for which, during their use, a number of irregularities were found within the external wall insulation installed with the ETICS. Selected defects are shown in Figure 1.

In order to determine the causes of the abnormalities and to assess the technical condition of the external walls, a performance of uncovering cut-outs of the insulation system in question was planned, together with taking samples of the structural elements by means of destructive methods.

The next step was to test the mass moisture of the construction elements directly under the insulation, the thermal insulation itself, the reinforcement layer, and the render, as well as the water absorption and capillary absorption of the render by means of the Karsten tube method. The research programme also included tests of interlayer adhesion. The research was summarised by numerical thermo-moisture simulations by means of the WUFI Pro 5.3. software.

Macroscopic tests of the insulation system were carried out. Microscopic tests were also performed using a device with 12× optical magnification.

The components of the insulation system in the samples were subjected to geometric measurements. In the case of the adhesive mortar and the reinforced layer with the thin-coat render system, the thickness measurements were carried out using a limit electronic calliper with a measuring range of 150 mm and a resolution of 0.01 mm (technical data of the limit measuring instrument in accordance with DIN 863-1 [29]).

Measurements of the mass moisture content of the wall substrates were carried out in 11 uncovering cut-outs of the insulation by means of an electronic moisture meter (moisture measurement on the surfaces of the structural wall). The measurements were performed with the use of a Testo 635-2 electronic instrument (Testo SE & Co. KGaA, Lenzkirch, Germany) with a material moisture probe. Measurements were taken at the locations of the uncovering cut-outs. The measuring accuracy of the device is ±1%.

Mass moisture tests were also carried out using the weighing dryer method on samples obtained from 6 boreholes of the structural substrate of the external walls (structural layer of the walls under the insulation—masonry made of silicate blocks). The obtained dimensions of the boreholes were as follows: diameter ca. 11.5 cm and height 9–11.5 cm. The extraction process was carried out using an electric drill rig equipped with a core drill. Three samples for mass moisture tests were taken from each borehole: from the outer surface, from the centre of the core, and from the inner surface of the borehole.

Mass moisture content tests were also carried out using the weighing dryer method on samples of the thin-coat render, the reinforced layer, and the thermal insulation layer. The test materials were obtained from samples of the thermal insulation system of the uncovering cut-outs.

The drying process of the samples was performed using a RADWAG MA 50.R electronic weighing and drying machine (Radwag, Radom, Poland). The test procedure was carried out in accordance with EN ISO 12570 [30]. The drying temperature was adapted to the type of sample material considered, according to the standard [30]. The accuracy of the weight measurement was 1 mg.

A water absorption test of the finishing layer of the ETICS was carried out by means of the Karsten tube method. The water absorption coefficient was determined by the amount of water absorbed (expressed in kg) per unit area of the regarded building envelope and per square root of time. The coefficient was determined according to the methodology given in EN ISO 15148 [31]. For each of the 10 samples, partial readings of the amount of water absorbed by the thin-coat render were recorded at time intervals of 30 s and 1, 2, 3, 4, 5, 7, 10, 15, 20, 30, and 40 min. For the cumulative amount of water absorbed after 40 min, the water absorption coefficient, expressed in kg/(m^2^∙h^0.5^), was calculated.

Laboratory tests of the water absorption of the facade render were also performed. These included 6 insulation samples obtained in the uncovering cut-outs. The water absorption tests of the rendering were carried out according to the procedure described in the European Assessment Document (EAD) [2].

Laboratory tests of the adhesion of the surface layer of the insulation system under consideration after ageing were performed. These included 2 insulation samples. The tests on the samples were determined according to the procedure described in [2].

### 2.1. Characteristics of the Object

The objects of this study are two multi-family residential buildings located in the Lesser Poland province of Poland. The buildings in question are multi-family residential buildings with 6 overground floors. The buildings are divided into two segments by an expansion joint. Entrances to the staircases are located on the northern facades. On the south side, the architecture features balcony recesses (upper floors) and terraces (ground floor). The buildings were erected using mixed technology. The walls of the foundations and at the ground floor are monolithic reinforced concrete. Above the ground floor, the walls are masonry made of silicate blocks. The facades were insulated with use of ETICS. The external walls are insulated with 15 cm thick panels of expanded polystyrene (EPS), with the addition of graphite, and with a heat conduction coefficient λ of 0.031 W/mK. Mineral wool boards were used locally for insulation (fire separation strips within the expansion joints). For the ground-floor walls, EPS-P (expanded polystyrene with increased moisture resistance) was used as thermal insulation. For the ETICS facade finishing, a silicone plaster with a textured structure was used.

### 2.2. Inspection of Uncovering Cut-Outs

To perform laboratory tests on the ETICS layers under consideration, and to determine their technical condition, 11 uncovering cut-outs were made in the insulation system on the buildings in question. The cut-outs were performed throughout the entire wall insulation system, i.e., the thermal insulation layer together with the thin-coat rendering and the adhesive that fastens the insulation to the substrate. The uncovering cut-outs were marked P1 to P5 (Building A) and P6 to P11 (Building B) (Table 1).

### 2.3. Numerical Modelling

Numerical calculations were carried out using the WUFI Pro 5.3 software, which enables simulation calculations of one-dimensional heat and moisture flow in multi-layered building partitions subjected to internal operating conditions and external weather conditions. The software performs one-dimensional calculations on building component cross-sections. The software performs heat and moisture transport calculations by means of the finite element method [26].

The simulations were carried out for an external wall model, the construction of which corresponded to the in situ external walls in the multi-family buildings analysed. As part of the calculations, the distribution of moisture content in the individual material layers (interstitial condensation phenomenon) was analysed.

For the development of the one-dimensional wall model, layers were adopted according to Table 2.

For the analysis of the one-dimensional moisture flow in the external wall under consideration, the climatic data of the meteorological station located in Kraków (database available through the WUFI Pro 5.3. software) were used. The information stored in the climatic database of the Kraków station was used as the basis for the external boundary conditions of the computer model. As internal boundary conditions of the tested partition, the humidity conditions were assumed in accordance with the provisions of the EN 15026 [32] standard (the temperature and relative humidity of the inside air were assumed in accordance with Annex C, assuming normal room occupancy). Material data for the construction of the models were taken based on the manufacturers’ declared performance properties and from the databases available within the software. The initial moisture content of the wall substrate (silicate blocks) was assumed based on in situ tests and measurements. A simulation period of six years was used to determine the distribution of moisture content in the analysed partition. The starting date of the computer simulation was set at 1 October, at 00:00.

## 3. Results

**Figure 2:**
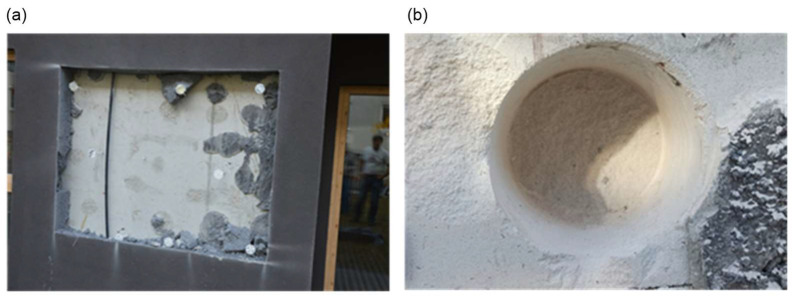
(**a**) Uncovering cut-out P6: Exposure of wall substrates. (**b**) Uncovering cut-out P2: Bore P2a in the wall construction substrate.

**Figure 3:**
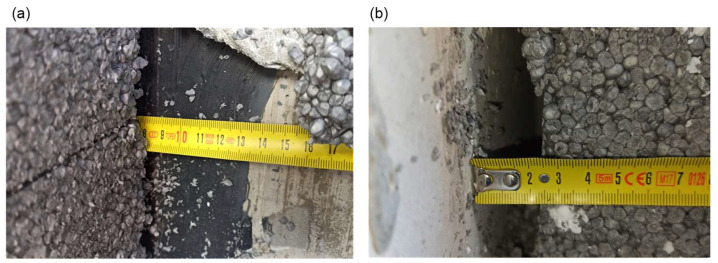
(**a**) Uncovering cut-out P9: Measurement of the thickness of the air void between the insulation and the wall substrate (ca. 2.5 cm). Thickness of the adhesive mortar exceeding 1 cm. (**b**) Uncovering cut-out P7: Measuring tape entering under the insulation at the joint of the polystyrene boards (no perimeter distribution of adhesive mortar).

**Figure 4:**
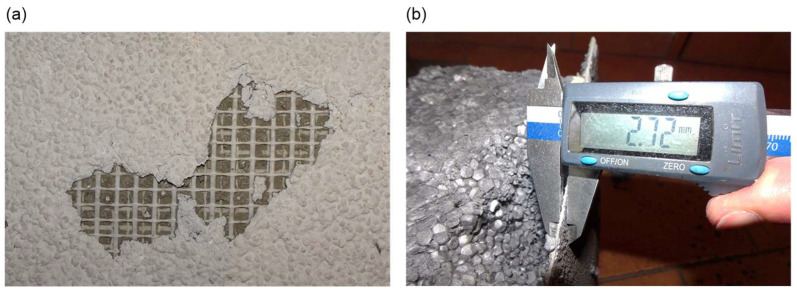
(**a**) Sample P9: Loss of facade render and a section of adhesive mortar exposing fibreglass mesh in the reinforced layer. (**b**) Sample P10: Measurement of the thickness of the thin-bed plaster and reinforced layer system (insufficient reinforced layer thickness, less than 3 mm).

**Figure 5:**
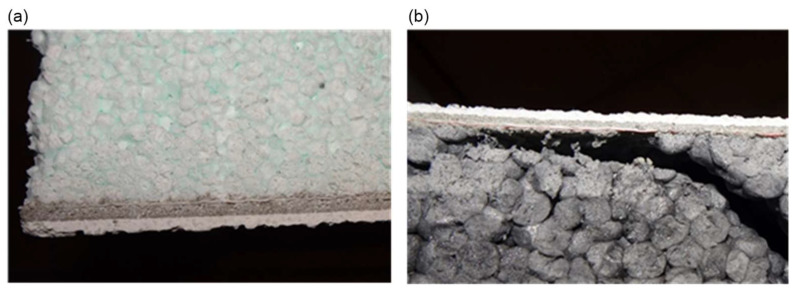
(**a**) Sample P1: View of the cross-section of the reinforced layer and the thin-coat plaster in the sample. The reinforcing mesh visible in the cross-section is not located in the middle of the thickness of the reinforced layer (deterioration of the mechanical properties of the reinforced layer). (**b**) Sample P11: View of the cross-section of the reinforced layer and the thin-coat plaster in the sample. The reinforcing mesh visible in the cross-section is not located in the middle of the thickness of the reinforced layer (deterioration of the mechanical properties of the reinforced layer).

**Figure 6:**
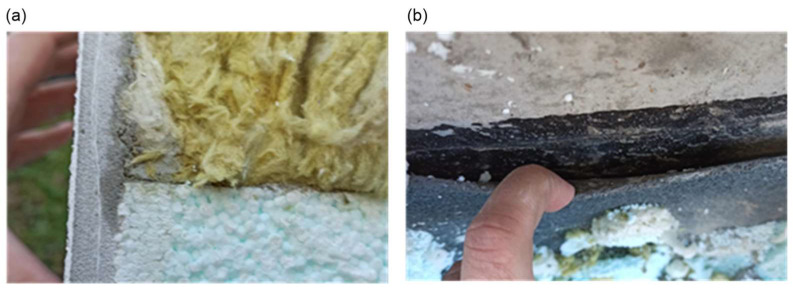
(**a**) Sample P1: Visually and organoleptically perceptible dampness of the mineral wool at the interface with the EPS-P insulation underneath the reinforced layer. (**b**) Uncovering cut-out P1: Lack of adhesion of bituminous membrane edge to the reinforced concrete wall substrate. No proper edge trim of the membrane with a pressure strip.

**Figure 7:**
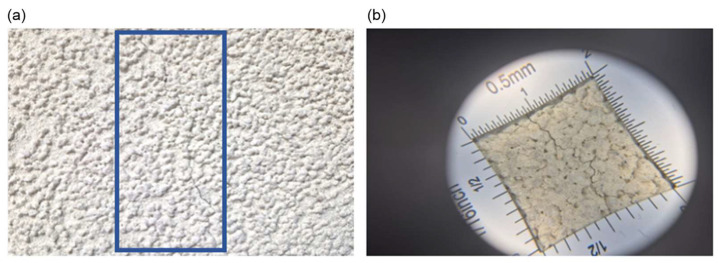
(**a**) Sample P9: Close-up of the surface of the facade plaster; visible cracks in the plaster; (**b**) Sample P9: Close-up of the surface of the facade plaster using an optical device; visible micro-cracks in the plaster.

Photographic documentation of the uncovering cut-outs of the insulation system in question is presented in Figure 2, Figure 3, Figure 4, Figure 5, Figure 6 and Figure 7. The descriptions of most of the photographs contain information on the noted anomalies.

**Figure 8:**
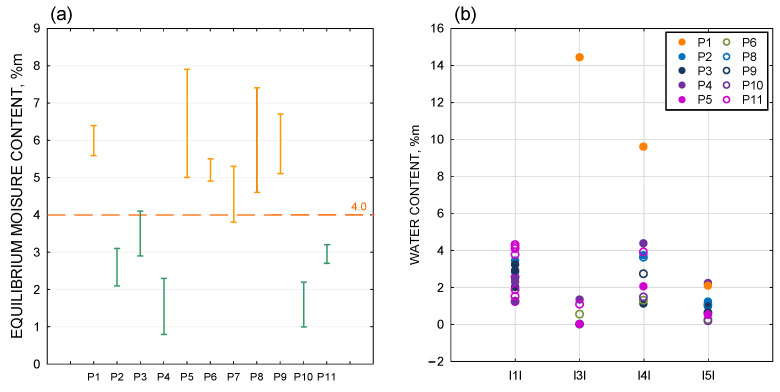
(**a**) Mass moisture content of the construction substrate obtained with the Testo 635-2 moisture meter (green—silicate block substrate, orange—reinforced concrete substrate). (**b**) Moisture content of the construction substrate, insulation layer, reinforced layer, and plaster, measured with a weighing dryer.

In the case of masonry walls made of silicate blocks, the measured moisture levels (Figure 8a), obtained with a Testo 635-2 moisture meter, ranged approx. 1–4%m. The silicate substrate tested in the uncovering cut-outs showed an acceptable moisture level. These results were very similar to the measurements made with the weighing dryer. The maximum differences between the two measurement methods did not exceed 2%, which is a very good result.

In the reinforced concrete walls, higher measured values (Figure 8a) of approximately 4–8%m were recorded. The results obtained for reinforced concrete walls were higher than the recommended maximum moisture content for the substrates specified in [33] (4%m—for concrete, ceramic, and silicate). However, the maximum moisture content of the substrate specified relates to the period prior to the fixing of the ETICS insulation boards. The measurement results obtained during the site visit do not necessarily reflect the moisture condition of the substrate prior to the insulation work. The buildings in question were in use at the time of the uncovering cut-outs and moisture meter measurements. Since the completion of the construction works, the moisture condition of the structural substrate may have changed due to the drying of the entire partition. The results of the moisture measurement do not provide any basis for claiming that the moisture content of the structural wall substrate in the analysed buildings was abnormal at the time when the insulation works were performed.

Tests carried out using a weighing dryer (Figure 8b) showed a low moisture content in the samples taken from the cores of the construction substrate (silicate blocks). Samples taken from the inner surface of the borehole showed values of 2.5–4.3%m, those from the centre 2.3–4.1%m, and those from the outer surface (the surface in contact with the insulation system) 1.2–2.0%m. The structural substrate of the external walls, made of silicate blocks, showed the permissible moisture level defined in [33] (4%m—concrete, ceramic, and silicate).

Testing of the thermal insulation samples showed mostly dry conditions of the samples. The mineral wool sample showed the highest moisture content (above 14%m) (Figure 8b).

Among the reinforced layer samples tested (Figure 8b), a high level of moisture was recorded in the same sample—over 9%m. The other samples of the reinforced layer showed results in the range of 1–4.5%m, i.e., the test showed an acceptable level of mass moisture content.

The thin-coat plaster samples tested (Figure 8b) showed a dry state, i.e., none of the results exceeded 3%m moisture by weight. The results obtained ranged from 0.2 to 2.2%m.

**Figure 9:**
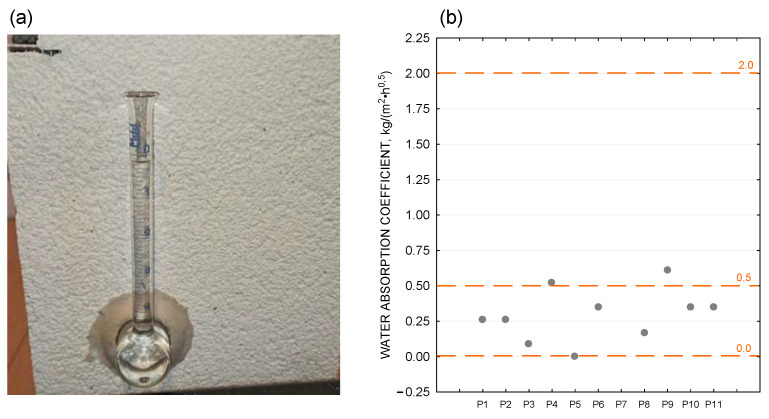
(**a**) Karsten tube water absorption coefficient test for plaster. (**b**) Water absorption coefficients determined using the Karsten tube method.

For the selected 10 ETICS insulation samples, a test was carried out to determine the water absorption coefficient using the Karsten tube method (Figure 9a). For the plaster tested, the results ranged from 0 to 0.6 kg/(m^2^∙h^0.5^) (Figure 9b). Considering the magnitude of the water absorption coefficient W_W_ of the protective coatings and the classification presented in DIN 4108-3 [34] (surface-absorbing layers: W_W_ ≥ 2.0 kg/(m^2^∙h^0.5^); impermeable layers: 0.5 kg/(m^2^∙h^0.5^) < W_W_ < 2.0 kg/(m^2^∙h^0.5^); surface hydrophobic layers: W_W_ ≤ 0.5 kg/(m^2^∙h^0.5^)), the facade plasters in the test samples can be classified as either impermeable or surface hydrophobic.

**Figure 10:**
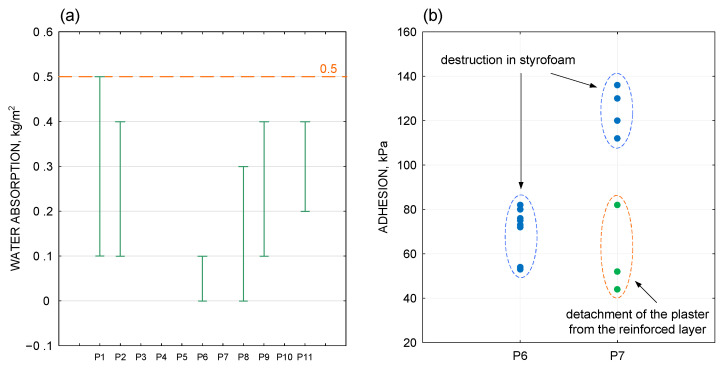
(**a**) Water absorption of plaster. (**b**) Adhesion of the surface layer.

According to the European Technical Assessment, the declared water absorption after 24 h is <0.5 kg/m^2^ for the surface layer of the adopted insulation system, where the finishing coat is plaster. In the analysed samples (Figure 10a), laboratory tests showed that the manufacturer’s declared water absorption after 24 h was met, with the exception of one sample, where the result was 0.5 ± 0.1 kg/m^2^. The increased water absorption result for this sample was due to its heavy exposure to precipitation moisture. It should be noted that this sample was taken from a location directly adjacent to the section exposed to splash water. Due to the increased exposure to rainwater, the silicone render at the indicated location showed increased water absorption. This was also confirmed by the dampness of the mineral wool observed during the extraction of the insulation sample from this uncovering cut-out.

For the two samples for which the adhesion of the surface layer was tested, partial adhesion values expressed in kPa are given, together with information on the location of the detachment of the surface layer (Figure 10b).

According to the provisions of document [2], all results of the surface layer adhesion test after ageing should meet the following conditions:

- Value should be at least 80 kPa at cohesive or adhesive rupture;
- If the result is less than 80 kPa, rupture must occur in the heat-insulating layer (100% cohesive fracture).

These conditions were fully met in the results of the first sample. For the second sample, the condition of adhesion after ageing was not fulfilled in two cases (out of seven carried out in total). The results obtained for this sample indicate faulty adhesion of the thin-coat render to the reinforced layer. The probable reason for the poor adhesion of the render to the reinforced layer is the variation in the preparation of the substrate before applying the render. It is presumed that the substrate for the facade layer was prepared incorrectly.

**Figure 11:**
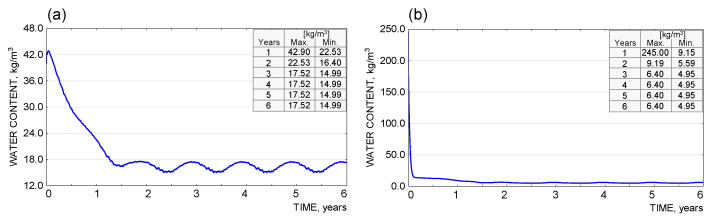
(**a**) Diagram of moisture content [kg/m^3^] in the wall construction layer (silicate blocks) |1|. (**b**) Graph of moisture content [kg/m^3^] in the adhesive mortar layer fixing the insulation to the wall substrate |2|.

**Figure 12:**
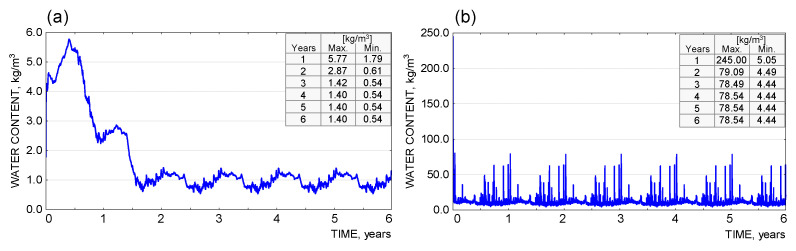
(**a**) Graph of moisture content [kg/m^3^] in the EPS 031 insulation layer |3|. (**b**) Graph of moisture content [kg/m^3^] in the reinforced layer |4|.

**Figure 13:**
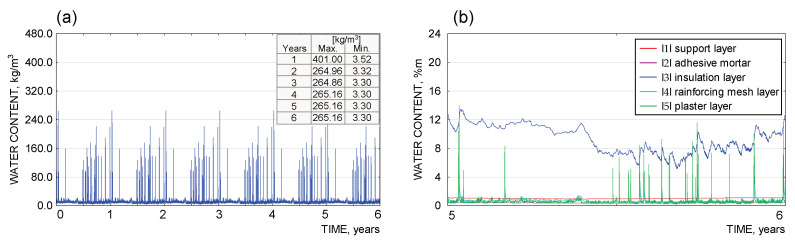
(**a**) Graph of moisture content [kg/m^3^] in the thin-coat plaster layer |5|. (**b**) Graph of moisture content [%m] in the layers of the envelope |1|–|5|, 6th year.

The results of the moisture analysis, carried out with WUFI Pro 5.3 software, are presented as graphs for each material layer separately (Figure 11, Figure 12 and Figure 13). The argument axis represents the time of analysis, while the value axis represents the quantity tested (moisture content in kg/m^3^, %m).

The results obtained from the simulation calculations in the WUFI Pro 5.3. software show that, for the partition under consideration, there is no risk of a continuous increase in condensate in the individual material layers. Due to the assumed presence of process moisture in the bonding layers (adhesive mortar, reinforced layer, facade render), an increased moisture content in the individual components was observed during the initial analysis period. The excess moisture evaporated over time, and the parameter under study stabilised (up to the end of the analysis, a periodic variation in moisture content was observed due to the variation in external conditions, typical of the temperate climate). In terms of avoiding the risk of interstitial condensation, the system adopted can be considered correct.

## 4. Discussion of the Results

The tests carried out on ETICS samples indicate the occurrence of numerous errors in the execution of insulation work that can affect the moisture status and durability of the building envelope. These include the following:

- Inappropriate method of bonding thermal insulation panels to the wall (lack of perimeter application of adhesive mortar, use of mixed method of bonding polystyrene panels to the substrate, excessive thickness of adhesive mortar resulting in the formation of wide air voids between the insulation and the structural substrate);
- No filling of the joints of the polystyrene boards (leaving the thermal insulation gaps at the joints of the EPS boards can lead to the phenomenon of interstitial condensation at the crevices between the boards);
- The lack of air-tightness of the insulation against the ingress of precipitation moisture (discontinuities in the plaster coating, unsealed enclosures for electrical outlets in the balcony recesses, lack of edge flashings between the balcony slabs and the side walls of the balcony recesses, unsealed connections between the insulation system layers and the external roller shutter cassettes, cracks in the plaster coating).

Mass moisture content testing of the wall substrate showed acceptable moisture levels within the masonry walls made of silicate blocks. In the case of reinforced concrete walls, locally elevated moisture levels were found. Tests carried out using a non-destructive method with a Testo 635-2 moisture meter gave similar results to those carried out using a weighing and drying machine.

Moisture tests carried out on the ETICS components in the analysed insulation samples showed a predominantly dry condition. Moisture testing of the reinforcement layer showed an acceptable level of moisture. Severe moisture was found in the sample taken at the interface between the mineral wool and the EPS-P insulation underneath the reinforced layer. This marked increase in dampness was the result of exposure of the insulation system in the ground zone to splash water from the surface of the ground. This example shows that some irregularities can contribute to significant dampness.

Capillary water absorption tests showed that the analysed silicone render could be classified as an impermeable and surface hydrophobic coating. The sample with the highest absorption showed mechanical micro-cracks in the plaster (Figure 7).

Tests of the water absorption of the facade plaster showed that the value declared by the manufacturer was mostly met. An increased value was obtained for the sample taken in the ground-floor zone, where the render is subjected to splash water from rainfall.

The test of the adhesion of the finish coat showed that the condition specified in [2] was not met for some of the test samples. This result indicates the possibility of a locally defective preparation of the substrate for the thin-coat render layer. In other cases, the required adhesion values for the same ETICS solution were achieved.

Simulation calculations of moisture transport in the analysed partition (masonry made of silicate blocks) showed that there was no continuous increase in condensation over the assumed analysis time (the influence of interstitial condensation on the observed anomalies was excluded).

## 5. Conclusions

A detailed analysis of the dampness of the ETICS was carried out for collective housing buildings. A number of deficiencies regarding the wall insulation system were highlighted. The irregularities that occurred with the insulation system were mainly related to execution errors. On the other hand, the technical condition of the facades in buildings is a result of the design and material solutions adopted, the quality of workmanship, and the use conditions of the insulation system.

The presented example is not an isolated case. This case study demonstrates the importance of proper design, dutiful construction, and subsequent use with proper moisture control involving the prevention of water ingress and condensation within the building.

This work contributes to the development and implementation of ETICSs with improved performance and durability.

## Figures and Tables

**Table 1:** Characteristics of the individual uncovering cut-outs.

No.	Cut-Out	Location of the Cut-Out		External Wall Construction Substrates	Thermal Insulation Layer
1	P1	Ground floor	Lower part of the wall, fire separation zone	Concrete	Mineral wool/ EPS-P polystyrene
2	P2	2nd floor	External wall outside balcony recesses	Silicate blocks	EPS polystyrene
3	P3	2nd floor	Pillar between balcony recesses	Silicate blocks	EPS polystyrene
4	P4	3rd floor	Near the expansion joint, fire separation zone	Silicate blocks	Mineral wool
5	P5	3rd/4th floors	At the height of the balcony floor slab between the 3rd and 4th floors, at the edge of the balcony floor slab flashing	Concrete	EPS polystyrene
6	P6	Ground floor	External wall near terrace recesses	Concrete	EPS polystyrene
7	P7	Ground floor	North wall of the staircase overhang	Concrete	EPS polystyrene
8	P8	Ground floor	West wall of the staircase overhang	Concrete	EPS polystyrene
9	P9	1st floor	Upper part of the wall	Concrete	EPS polystyrene
10	P10	2nd floor	Pillar between balcony recesses	Silicate blocks	EPS polystyrene
11	P11	2nd floor	Lower part of the wall, near northwest corner of the building	Silicate blocks	EPS polystyrene

**Table 2:** Layered structure of the wall model.

Layer No.	Material	Thickness [cm]
|1|	Silicate blocks masonry wall	24.00
|2|	Adhesive mortar fixing the insulation to the substrate	1.00
|3|	EPS polystyrene	15.00
|4|	Reinforcing mesh layer	0.03
|5|	Silicone plaster	0.015

## Data Availability

The original contributions presented in this study are included in the article. Further inquiries can be directed to the corresponding author.
